# Differential diagnosis between somatic and psychiatric disorders based on laboratory tests integrated with neuroimaging

**DOI:** 10.1192/j.eurpsy.2025.237

**Published:** 2025-08-26

**Authors:** K. Krysta

**Affiliations:** Department of Rehabilitation Psychiatry, Medical University of Silesia, Katowice, Poland

## Abstract

**Abstract:**

The differentiation between somatic and psychiatric disorders presents a significant challenge in clinical practice due to overlapping symptomatology and complex etiologies. This abstract proposes an integrated approach utilizing laboratory tests and neuroimaging techniques to enhance diagnostic accuracy and improve patient outcomes. Somatic disorders, characterized by physical symptoms with identifiable organic causes, often mimic psychiatric conditions, leading to misdiagnosis and inappropriate treatment. Laboratory evaluations play a crucial role in ruling out medical conditions that may present with psychiatric-like symptoms. Thyroid function tests, vitamin B12 level assessments, and screening for infections are vital in this process. Routine blood tests further aid in detecting systemic conditions contributing to psychiatric presentations. Neuroimaging techniques offer visual insights into brain structure and function, facilitating the differentiation between psychiatric and neurological conditions. Structural imaging modalities like MRI and CT scans can reveal abnormalities such as tumors or lesions. Functional imaging, including PET and SPECT scans, assesses cerebral metabolism and blood flow, identifying anomalies associated with specific psychiatric disorders. Integrating laboratory tests with neuroimaging significantly enhances diagnostic precision. In suspected dementia cases, for instance, laboratory tests can exclude metabolic or infectious causes, while neuroimaging can identify characteristic patterns of Alzheimer’s disease or vascular dementia. This combined approach ensures a thorough evaluation, reducing misdiagnosis and facilitating targeted treatment strategies. Clinical studies have demonstrated the efficacy of this integrated approach in improving diagnostic accuracy for conditions like major depressive disorder and bipolar disorder. Functional neuroimaging has also proven instrumental in distinguishing between psychiatric disorders and neurological conditions such as epilepsy. Despite its advantages, this approach faces challenges, including cost, availability, and the need for standardized interpretation protocols. Future directions include advancements in neuroimaging accessibility, research into novel biomarkers, and the integration of artificial intelligence for enhanced diagnostic accuracy and personalized treatment planning. In conclusion, integrating laboratory tests with neuroimaging represents a significant advancement in the differential diagnosis of somatic and psychiatric disorders. This comprehensive approach facilitates accurate diagnosis, ensuring patients receive appropriate and effective treatment, ultimately leading to improved patient outcomes.

**Disclosure of Interest:**

None Declared

